# Emotional priming depends on the degree of conscious experience

**DOI:** 10.1016/j.neuropsychologia.2017.10.028

**Published:** 2019-05

**Authors:** Michael Lohse, Morten Overgaard

**Affiliations:** a Department of Physiology, Anatomy and Genetics, University of Oxford, OX1 3PT Oxford, United Kingdom; b Cognitive Neuroscience Research Unit, CFIN, Aarhus University, Noerrebrogade 44, Building 10G, 8000 Aarhus, Denmark

**Keywords:** Graded awareness, Perceptual awareness scale, Emotion, Priming, Faces

## Abstract

Most experiments in consciousness research assume that awareness is a dichotomous 'either/or' phenomenon. However, participants can distinguish multiple levels of subjective experience of simple features (colour, shape etc.), which correlate with their performance in different tasks. As experiments showing multiple levels of perceptual awareness question the widespread idea that many forms of perception can occur unconsciously, we investigated emotional priming combined with methods able to measure small variations in subjective experience.

We show awareness of emotional faces is gradual rather than dichotomous, and that the effects of emotional priming are predicted by the level of perceptual awareness of emotional faces, with no effects when reported unseen.

The results question how much unconscious perceptions can influence behaviour. As priming is one of the most well-established phenomena believed to occur unconsciously, the results expand the growing body of evidence that questions the contributions of unconscious processing on behaviour.

## Highlights

• Emotional priming is considered fundamental evidence for unconscious perception.
• Emotional priming strength is predicted by graded perceptual awareness levels.
• Emotional priming with faces is not effective when faces are reported as unseen.
• Facial expression recognition increases gradually with perceptual awareness levels.
• Perceptual awareness of faces increases gradually with duration of face stimuli.

## 1 Introduction

Subliminal priming is a modulation of behaviour caused by a presented, but unexperienced, stimulus (Bar and Biederman, 1998, McConnell et al., 1958). Subliminal visual priming with emotional stimuli has been suggested to affect the evaluation of the emotional content of subsequently presented stimuli – a phenomenon that has been described as subliminal emotional (affective) priming (Hermans et al., 2003; Kunst-Wilson et al., 1980; Murphy and Zajonc, 1993; Tamietto and De Gelder, 2010). Subliminal priming is typically considered to be one of the strongest pieces of evidence for the existence of unconscious mental states (Dolan, 2002). However, it is based on the assumption of a perceptual awareness threshold. Recent evidence has challenged this assumption, suggesting that perceptual awareness of, at least, simple stimulus features are more exhaustively described by a graded account (i.e., multiple levels) of awareness, rather than a dichotomous account where a stimulus can either be “seen” or “not seen”, relative to a threshold (Ramsøy and Overgaard, 2004, Overgaard et al., 2006, Sandberg et al., 2010, Windey et al., 2014, Wierzchoń et al., 2014, Overgaard and Sandberg, 2012). Evidence supporting a gradual account of visual consciousness comes from experiments using a combination of forced-choice and subjective reports in visual masking paradigms (Ramsøy and Overgaard, 2004, Overgaard et al., 2006, Sandberg et al., 2010, Windey et al., 2014, Wierzchoń et al., 2014, Overgaard and Sandberg, 2012, Sandberg and Overgaard, 2015, Lähteenmäki et al., 2015). Nevertheless, the topic is still controversial, and to a high degree, existing evidence suggests that relatively small manipulations of the experimental procedure will generate rather different results (Sergent and Dehaene, 2004, Windey et al., 2014, Ramsøy and Overgaard, 2004). One cornerstone in this debate is the phenomenon blindsight, originally coined by Larry Weiskrantz (Weiskrantz et al., 1974, Weiskrantz, 1986). Blindsight has for decades played a central role in the study of unconscious perception and has been studied extensively using a creative multitude of experimental paradigms (e.g. Azzopardi and Cowey, 1997; Kolb and Braun, 1995; Persaud and Lau, 2008; Trevethan et al., 2006) as well as blindsight patients. Whereas most experiments with blindsight patients have suggested that this syndrome is a perfect example of completely unconscious perception (Weiskrantz, 2009), more recent experiments using gradual reports indicate that graded levels of subjective experience predict objective performance (Overgaard et al., 2008, Overgaard, 2011; Stoerig and Bath, 2001; Mazzi et al., 2016). In relation to affective processing, blindsight patients have also been reported to respond above chance level to visually presented emotional information presented in their cortically damaged visual field (Celeghin et al., 2015). How much, and in which way, blindsight relates to the discussion of unconscious perception is still not fully clear (Azzopardi and Cowey, 1997, Kolb and Braun, 1995; Marzi et al., 2004), and a reevaluation has been suggested in recent time (Brogaard, 2011; Overgaard and Mogensen, 2015).

Currently, priming still stands as unrefuted evidence for unconscious perception, and, accordingly, remains one of the strongest lines of evidence for unconscious perception. Yet, it remains to be seen whether an investigation of emotional priming using exhaustive subjective methods would support the gradual or the dichotomous view of conscious experience. Evidence that priming only works when there is some level of subjective experience, and gradually increases with increased awareness, would strongly challenge the otherwise widespread idea of unconscious visual perception. Such a result would be in accordance with a recent study showing a dependence of perceptual awareness level on the ability to discriminate emotional stimuli (Lähteenmäki et al., 2015). On the other hand, evidence that priming works in the complete absence of experience, regardless of subjective methods, would suggest that consciousness is not gradual under all conditions.

Here we show that perception of static emotional faces is better described by multiple graded levels awareness and, importantly, emotional priming effects on subsequent emotional judgements are continuously predicted by how clearly the priming stimulus is seen, with a minimal level of awareness required for both priming and facial expression recognition. This finding challenges the traditional conception of unconscious facial expression recognition and emotional priming. It suggests that effects traditionally regarded to be caused by unconsciously perceived stimuli might more adequately be described as being caused by a degraded level of perceptual awareness of the stimulus.

## 2 Experimental methods

All experiments were carried out in accordance with Danish law, and were approved by the scientific ethical committee in Region Midtjylland, Denmark.

### 2.1 Participants

Fourteen healthy, Danish, participants took part in the experiment (6 male, 8 female), after giving written informed consent. Participants were between 20 and 30 years old and had normal or corrected-to-normal vision.

### 2.2 Stimuli and task

**Fig. 1:**
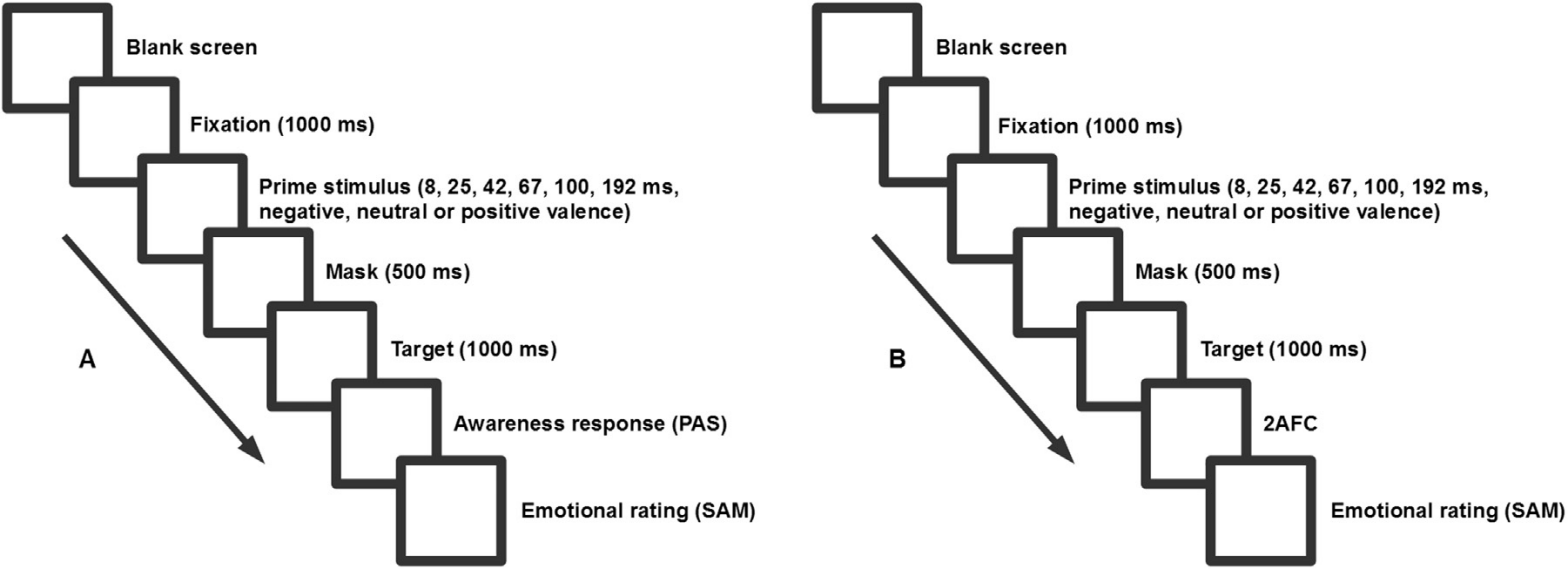
Experimental paradigm.

Stimuli were presented on a 21 in. 120 Hz CRT monitor, and participants responded on a custom built external keyboard. Stimuli comprised static greyscale images displaying 24 neutral faces and 48 emotional faces (24 positive and 24 negative valence) from the “Karolinska directed emotional faces” (Lundqvist et al., 1998), as well as a 24 white Chinese symbols (which participants did not understand the meaning of). At the beginning of each trial a white fixation cross was presented in the centre of the screen. All stimuli were presented on a black background. The paradigm was presented using OpenSesame (Mathôt et al., 2012). Prime stimulus durations (~ 8 ms (8.333 ms), ~ 25 ms (24.99 ms), ~ 42 ms (41.66 ms), ~ 67 ms (66.66 ms), ~ 100 ms (99.99 ms), ~ 192 ms (191.66 ms)) were measured by counting the onset and offset of frames on the CRT monitor using the OpenSesame and the internal clock of the computer. Prime stimuli durations were subsequently confirmed as being the intended durations using the internal clock measurements. For each part of the experiment we presented each duration eight times for each valence giving us 144 trials per part for a total of 288 trials. This meant 144 PAS judgements, 144 2AFC and 288 emotional ratings per participant. See Fig. 1 for procedure description.

We asked participants for three types of responses during the trials: (1) How aware they were of the prime stimulus presented. This was measured with introspective perceptual awareness reports using the *perceptual awareness scale* (PAS) (Ramsøy and Overgaard, 2004). (2) How accurately participants could recognise the priming stimulus. This was done using a 2AFC, where the distractor in the 2AFC was a face with same identity as the prime stimulus, but with one of the two alternate facial expression chosen randomly. Participants were asked to select the prime stimulus which were presented, or guess if they did not know what prime stimulus had been presented. (3) An emotional rating of a non-sense symbol, presented by the end of each trial, using the valence section of the *self-assessment manikin* (SAM) (Lang, 1980). The experiment was divided into two separate counter balanced parts: (A) Participants report awareness of prime stimulus and emotional rating of a non-sense symbol, (B) Participants are asked in a 2AFC which prime stimulus was presented, and perform an emotional rating of a non-sense symbol. We carried out the experiment in these two counter balanced parts in order to avoid contamination between the scales and to keep the decisions about the prime stimuli as close in time to the presentation of the prime stimuli as possible.

PAS allows the participants to report the perceptual awareness of a visual stimulus with four values: *No experience (1)*, *brief glimpse (2)*, *almost clear experience (3)*, and *clear experience (4)*. PAS was developed in a study by Ramsøy and Overgaard (2004), where participants were asked to describe the clarity of briefly presented visual stimuli of simple geometric shapes, using a scale they were free to create themselves. This was done in order to create a perceptual awareness scale based on perceptual awareness levels participants would naturally use. All participants tested (> 5) ended up using a four-point scale closely approximating the four PAS levels used in the current study (Ramsøy and Overgaard, 2004, Sandberg and Overgaard, 2015).

The valence section of SAM is a graphical nine-point scale, where participant can report emotional content ranging from very negative through neutral to very positive (Lang, 1980). For quantitative analysis these values were considered as continuous where the responses were translated into numerical values (−4 to 4), with −4 being very negative, 0 being neutral, and 4 being very positive.

Participants performed 72 pilot trials (PAS + SAM reports), before part A of the experiment, and 10 pilot trials (2AFC + SAM) before part B of the experiment, in order for the participants to get acquainted with the paradigm (Sandberg and Overgaard, 2015).

The experiment consisted of two parts. (A + B) Participants initiated each trial by pressing 'space' during the presentation of a blank screen. Once the trial was initiated they were presented with a white fixation cross on a black background. After a 1000 ms the prime stimulus was presented. The prime stimuli consisted of a neutral or emotional face presented at either 8, 25, 42,67, 100, or 192 ms. Immediately following the prime stimulus, a mask displaying a neutral female face was presented for 500 ms. Subsequently, an irrelevant neutral target symbol (i.e., Chinese symbol) was presented for a 1000 ms. Participants then had to respond (A) how aware they were of the prime stimulus, and subsequently make a valence judgement of the target irrelevant neutral stimulus, or (B) respond which face had been shown as the prime stimulus in a 2AFC, and subsequently make a valence judgement of the irrelevant neutral target stimulus.

### 2.3 Data analysis

All analyses were carried out in Matlab R2015a. We analysed all data using a mixed-model approach.

From a statistical point of view, PAS can be interpreted in different ways (Sandberg and Overgaard, 2015). PAS has previously been analysed as an interval scale assuming a linear relationship between the different levels of awareness (Sandberg et al., 2010, Sandberg et al., 2011, Schwiedrzik et al., 2011, Windey et al., 2013). Furthermore, by assessing proposed neural signatures of awareness - the visual awareness negativity and late positivity – Tagliabue et al. (2016) found a near linear increase in the visual awareness negativity, as well as the following neural component, late positivity, with increasing PAS levels. However, PAS has also previously been treated as an ordinal scale (Wierzchoń et al., 2014). We therefore chose to analyse the results separately using both linear and ordinal analyses. For the linear analysis the responses were assumed to have equal intervals between them and were translated into numerical values (1–4), with 1 representing *No experience* and 4 representing *clear experience.* For the ordinal analysis, priming effects were estimated separately for each PAS level.

The mixed-model approach consisted of modelling within-subject responses with (1) multinomial (PAS distribution as a function of prime duration), (2) binomial (facial expression recognition accuracy as function of awareness), (3) linear (emotional priming strength as a function of awareness) regressions or (4) ordinal (emotional priming strength as a function of awareness report) regressions. For 1 and 2, we bootstrapped the within-subject results using 10.000 samples with replacement. This allowed us to estimate 95% bootstrapped confidence intervals, and statistically infer significant differences at a group level. We considered no overlap between the 95% CI of the different conditions as a significant difference. For 3, we carried out a mixed-model linear regression with random slope and random intercept by running one-sided (in the direction of the prime stimulus valence) *t*-tests on the normalised slope (*β1)* value and intercept of within-subject linear regressions. Each point in the within-subject linear regressions corresponds to the mean emotion score over the mean PAS response at each presented prime stimulus duration. For 4, we carried a first level (within-subject) analysis using Spearman's *Rho* for ordinal correlation between PAS report and emotion score. The second level analysis consisted of a *t*-test of the Spearman's *Rho* values derived from the first level analysis. The linear and ordinal mixed-model regression and correlation were carried out to test the hypothesis that perceptual awareness predicted the strength of emotional priming (slope (β1) or Rho) in the direction of the emotional prime valence, as well as the effects of emotional priming when participants had no experience of the prime stimulus (intercept/No experience report). To account for potential emotional effects related to simply increasing stimulus duration and potential effects of increasing awareness of faces in general, we calculated the emotion score for the linear analysis by subtracting the SAM value of the neutral priming stimuli from the SAM value of the emotional priming stimuli at each stimulus duration. For the ordinal analysis we accounted for such bias by testing the emotional priming strength at each PAS level against the priming effect when presenting neutral primes.

## 3 Results

We investigated whether the perception of static emotional faces were better described by a graded account of awareness, compared to a dichotomous account of awareness. Furthermore, we assessed whether emotional priming effects and facial expression recognition were related to how clearly a priming stimulus was seen.

### 3.1 Perceptual awareness of faces

We assessed whether participants needed multiple levels of perceptual awareness or only two levels (i.e., *no experience* or *clear experience*) to describe their perceptual awareness of briefly presented (8–192 ms) emotional and neutral faces. We tested this by inferring whether people regarded the prime stimuli as becoming gradually more visible (i.e., all PAS values required for an exhaustive measure) or simply shifted from a “not seen” to a “seen” condition (i.e., only *no experience* and *clear experience* would be used).

We predicted that if multiple levels of perceptual awareness are needed to understand perceptual awareness of faces, then there would be prime stimulus durations where intermediate perceptual awareness reports (i.e., *brief glimpse* and/or *almost clear*) would be significantly more likely to be chosen, compared to *no experience* and *clear experience*.

**Fig. 2:**
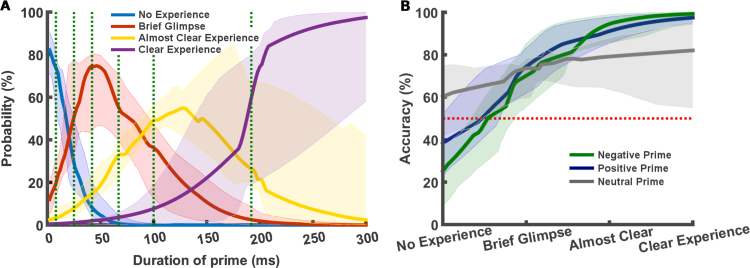
Perceptual awareness of faces and facial expression recognition A) Probability of PAS reports between 2 and 300 ms. Responses were recorded at 8 ms, 25 ms, 42 ms, 67 ms, 100, and 192 ms (vertical green stippled lines). The intermediate points are estimated with a mixed-model multinomial logistic regression (*n* = 14). B) Facial expression recognition accuracy as a function of mean (for each duration) PAS level. Red stippled line indicates chance level. Shaded areas represent 95% bootstrapped confidence intervals (*n* = 14). (For interpretation of the references to color in this figure legend, the reader is referred to the web version of this article)..

We found that both *brief glimpse* and *almost clear experience* could be significantly separated from *no experience* and *clear experience* (Fig. 2A, Table 1). Table 1 summarises the statistical differences in response probabilities of the four PAS levels at the presented durations. *No experience* was significantly more like than any other perceptual awareness responses, when the prime stimulus was duration 8 ms. *Brief glimpse* was significantly more likely than any other perceptual awareness responses, when the prime stimulus duration was 25 and 42 ms. *Almost clear* was significantly more likely to be reported compared to any other perceptual awareness responses, when the prime stimulus duration was 100 ms. Finally, *clear experience* was significantly more likely to be reported compared to *no experience* and *brief glimpse*, but not *almost clear,* when the prime stimulus duration was 192 ms.

**Table 1:** PAS response probability. The top of the table shows the median probabilities of choosing a given PAS response when stimuli were presented at either 8, 25, 42, 67, 100, or 192 ms. The bottom part of table illustrates the *p*-values from statistical tests (Wilcoxon signed rank test) of all combinations of possible PAS responses at all presented durations. Significant values (*p* < 0.05) are highlighted in bold.

	***PAS/Duration***	**8 ms**	**25 ms**	**42 ms**	**67 ms**	**100 ms**	**192 ms**
**Median**	**No experience (NE)**	75.3	26.79	14.58	6.55	4.46	3.57
**PAS response**	**Brief glimpse (BG)**	20.54	63.39	67.26	47.32	28.57	9.52
**probability (%)**	**Almost clear (AC)**	3.87	9.52	16.07	34.22	43.75	40.48
	**Clear experience (CE)**	0.3	0.3	2.08	11.91	23.21	46.43
							
	**NE > BG**	**0.0006**	0.9921	0.9996	0.9996	0.9998	0.9883
	**NE > AC**	**0.0001**	**0.0200**	0.8289	0.9957	0.9993	0.9994
	**NE > CE**	**< 0.0001**	**0.0005**	**0.0068**	0.6348	0.9858	0.9991
	**BG > NE**	0.9996	**0.0092**	**0.0005**	**0.0006**	**0.0005**	**0.0127**
	**BG > AC**	**0.0002**	**0.0001**	**0.0004**	0.0923	0.9585	0.9995
**Wilcoxon test:**	**BG > CE**	**0.0002**	**< 0.0001**	**< 0.0001**	**0.0143**	0.2767	0.9962
**p-value**							
	**AC > NE**	0.9999	0.9829	0.1816	**0.0060**	**0.0010**	**0.0007**
	**AC > BG**	1.0000	1.0000	0.9997	0.9141	**0.0450**	**0.0007**
	**AC > CE**	**0.0156**	**0.0010**	**0.0002**	**0.0096**	**0.0220**	0.6370
	**CE > NE**	1.0000	1.0000	0.9941	0.4043	**0.0146**	**0.0012**
	**CE > BG**	1.0000	1.0000	1.0000	0.9872	0.7350	**0.0043**
	**CE > AC**	1.0000	1.0000	1.0000	0.9917	0.9801	0.3746

In order to illuminate if *almost clear* and *clear experience* responses were fully separable, as well as at what stimulus durations each of the four PAS reports are needed, we used a multinomial regression model to estimate PAS responses from 2 to 300 ms with 2 ms resolution, based on the actual responses given at the presented prime stimulus durations (8.33–191.66 ms).

By modelling the PAS reports with a mixed-model multinomial regression, we found that the probability of choosing each of the four PAS levels were significantly more likely (no overlap of 95% confidence intervals (CI)) than any other PAS levels at unique prime stimulus durations (Fig. 2A and Fig. S2). *No experience* was significantly more likely than any other PAS levels when prime stimulus duration were between 2 and 12 ms. *Brief glimpse* was significantly more likely than any other PAS levels when prime stimulus duration were between 30 and 66 ms. *Almost clear experience* was significantly more likely than any other PAS levels when prime stimulus duration were between 104–128 ms. *Clear experience* was significantly more likely than any other PAS levels when prime stimulus duration were 274 ms or longer. Though individual variability in PAS reports is evident, no participant exclusively used *no experience* and *clear experience*, and only two participants did not use all four PAS levels (Fig. S1).

The results of the direct comparison of perceptual awareness reports at presented durations, as well as the multinomial model predictions of perceptual awareness responses of prime stimuli at durations between the presented prime stimulus durations, support the hypothesis that faces (i.e., neutral and emotional faces) presented at short duration can be perceived with multiple graded levels of awareness. The direct comparisons of PAS responses at presented stimulus durations (Table 1) suggest that *almost clear* and *clear experience* responses might belong to the same category, suggesting that three levels of perceptual awareness can adequately describe perceptual awareness of faces at short durations. However, the multinomial model predictions (Fig. 2A) support the idea that all four levels of PAS are needed. This last interpretation, based on bootstrapped results derived from the multinomial regression, are only tentative, given the fact that non-parametric bootstrapped confidence intervals are less reliable with small samples, as is the case with the current study (i.e., *n* = 14) (Chernick, 2008).

Furthermore, using a mixed-model binomial regression, we assessed if the levels of perceptual awareness were associated with facial expression recognition accuracy (Fig. 2B). We found that facial expression recognition accuracy gradually increased as a function of mean PAS levels for all prime stimuli valences. However, facial expression recognition accuracy as a function of perceptual awareness appears to increase slower for neutral faces compared to emotional faces. Wilcoxon tests (Accuracy (%) > Chance (50%)) showed that for PAS level *No experience,* accuracies were not significantly above chance level, for any of the three valences. For *brief glimpse, almost clear* and *clear experience*, accuracy were significantly above chance for all stimulus valences. Table 2 summarises the predicted accuracy level with statistical inference at each of the PAS levels for each valence, based on the binomial regression. By modelling the facial recognition accuracy between the four reportable PAS levels, we could further probe the development of facial expression recognition accuracy as a function of perceptual awareness level (Fig. 2B). Facial expression recognition became significantly greater than chance (no overlap of 95% CI with 50% accuracy) at a PAS value of 1.78 (slightly before *brief glimpse*) for negative primes, 1.71 (slightly before *brief glimpse*) for positive primes, and 1.52 (between *no experience* and *brief glimpse*) for neutral primes. These results suggests that minimal awareness (i.e., *brief glimpse*) is needed for facial expression recognition to occur.

**Table 2:** Face expression recognition accuracy as a function of PAS level. The table shows the median accuracy (across participants) for each of the three stimulus valences at each of the inferred PAS levels, based on the binomial regressions. The *p*-value of Wilcoxon signed rank tests, testing the accuracy against chance level is shown in parenthesis. Significant values (Wilcoxon test, Accuracy (%) > Chance (50%), *p* < 0.05) are highlighted in bold.

**Valence/PAS**	**No experience**	**Brief glimpse**	**Almost clear**	**Clear experience**
**Positive**	38.28 (0.95)	**74.41 (0.0015)**	**91.13 (0.0001)**	**97.49 (< 0.0001)**
**Neutral**	60.32 (0.18)	**73.67 (0.0020)**	**79.16 (0.0006)**	**82.05 (0.0015)**
**Negative**	25.28 (0.99)	**70.14 (0.0034)**	**94.53 (< 0.0001)**	**99.32 (< 0.0001)**

### 3.2 Perceptual awareness and emotional priming

Having demonstrated that the experimental stimuli (faces) gave rise to graded levels of awareness, we were able to test how levels of perceptual awareness relate to the effect of emotional priming.

**Fig. 3:**
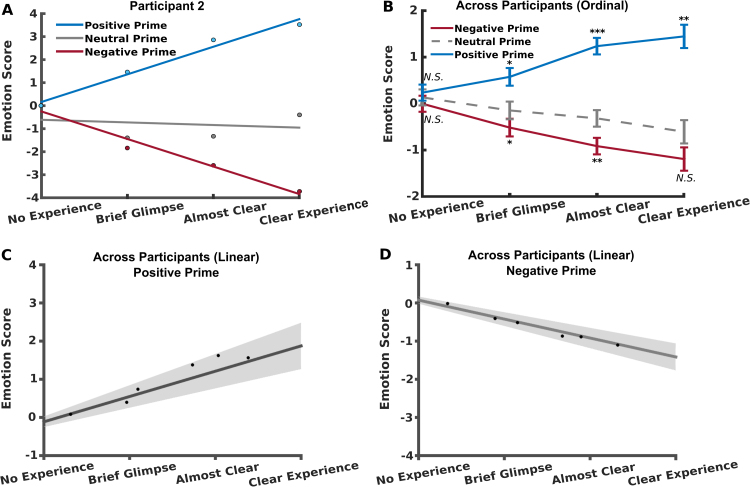
Level of perceptual awareness and emotional priming effect. (A) Illustration of emotional priming effect as a function of PAS report for an example participants. (B) Ordinal analysis of priming strength as a function of PAS report across participants. Asterisks indicate significance levels of *t*-tests between emotional priming condition (positive or negative) vs neutral priming condition for each PAS level - *: *p* < 0.05, **: *p* < 0.01, ***: *p* < 0.005, *N.S*: *p* > 0.05. (C) Mixed-model linear regression of positive priming effect as a function of perceptual awareness. D) Linear regression of negative priming effect as a function of perceptual awareness. Points in C and D represent mean PAS (group) over mean emotion score (group) per duration. Shading and error bars indicate standard error of the mean of intersection and slope (*n* = 14).

First we used a mixed-model linear regression to assess the relationship between PAS level and emotional priming strength. The mixed-model analysis was performed by estimating the *β1 (*slope) of the relationship between PAS level and emotional priming strength (first/subject level), followed by a statistical comparison of all the subject specific β1's against zero (second/group level). We found that perceptual awareness (as measured by PAS) predicted positive emotional priming effects (as measured by self-assessment manikin (SAM) rating of neutral irrelevant stimulus), in the direction of the valence, group mean ± standard deviation *β1* = 0.67 ± 0.33, *t*(13) = 7.6361, *p* < 0.0001 (Fig. 3C, see Figs. S2 and S4 for within-subject model fits).

Likewise, we found level of perceptual awareness predicted effects of negative emotional priming, in the direction of the valence, group mean ± standard deviation *β1* = −0.64 ± 0.36, *t*(13) = −6.5543, *p* < 0.0001 (Fig. 3D, see Figs. S3 and S4 for within-subject model fits).

In the analysis above, we subtracted effects induced by priming with neutral faces (control prime), allowing us to control for any potential effects related to increasing perceptual awareness of faces in general. However, emotional judgements of the target was unaffected by awareness state when preceded by neutral primes, *t*(13) = −2.1104, *p =* 0.053*,* and a comparison between the regression with emotional priming strength and perceptual awareness for neutral primes and emotional primes showed similar results to the ones reported above with *t*(13) = 4.8162, *p* < 0.0001 and *t*(13) = −2.9038, *p* = 0.006, for positive primes and negative primes, respectively.

Furthermore, we found no significant effect of emotional priming at the intercept with the y-axis (i.e., no experience), for neither positive priming, *t*(13) = −0.8672, *p* = 0.7992 nor negative priming *t*(13) = 0.8531, *p* = 0.7955.

When analysing the PAS as an ordinal scale, we replicated the results from the linear analysis. The ordinal analysis showed no significant (Bonferroni corrected *α* = 0.0125) priming was present when participants reported a stimulus as no experience, for both negative < neutral prime, *t*(13) = −0.8325, *p* = 0.21, and positive > neutral prime, *t*(13) = 0.7601, *p* = 0.23. A significant priming effect was present when participants reported a brief glimpse of the stimulus for both negative < neutral prime, *t*(13) = −3.557, *p* = 0.0018, and positive > neutral prime, *t*(13) = 2.816, *p* = 0.0073. Emotional faces were also significantly priming emotional responses when participants reported the emotional positive stimuli as almost clear, *t*(13) = 4.261, *p* = 0.0005 and clear experience, *t*(9) = 3.324, *p* = 0.0051. For negative emotional faces, we found that participants were significantly primed by the emotional face when reporting the prime stimulus as almost clear, *t*(13) = −3.4096, *p* = 0.0023, but not when reporting the stimulus as no experience, *t*(10) = −1.2498, *p* = 0.12. This latter result, however, could be attributed to a combination of the priming with neutral primes showing a slight negative bias, and the fact that three participants chose not to report any negative or neutral stimuli as clear experience, decreasing the *n* of the negative < neutral for clear experience test to 11 participants. A mixed-level ordinal analysis, with the within-subject summary statistic being Spearman's *Rho* for ordinal correlation, we found that the emotion score after positive and negative primes were significantly correlated with PAS reports: Negative prime, *Rho* = −0.49 ± 0.60, *t*(13) = −3.097, *p* = 0.0042, and positive prime, *Rho* = 0.53 ± 0.60, *t*(13) = −3.097, *t*(13) = 3.3003, *p* = 0.0029. However, the emotion score following neutral prime did not show a significant correlation with PAS reports after Bonferroni correction, *Rho* = −0.34 ± 0.50, *t*(13) = 2.5349, *p* = 0.03 (i.e., *α* = 0.0167) (Fig. 3A, B).

We calculated the Bayes factor to statistically test the evidence supporting the null hypothesis (no priming effect when prime stimulus is reported as not experienced). We calculated the Bayes factor (BF) of the null hypothesis using the method outlined in Dienes (2014). The predicted effect sizes of subliminal emotional priming effects used in our bayesian analysis were taken from a seminal emotional priming study by Murphy and Zajonc (1993). A BF less than 0.33 is by convention considered as statistical evidence for the null hypothesis (no priming effect when the priming stimulus is reported as *no experience*) (Jeffreys, 1939/1961, Jeffreys, 1939/1961). We found that the BF for the emotional priming effect when prime stimuli were reported as not experienced (intercept in mixed linear analysis) was 0.09 for positive prime stimuli and 0.07 for negative prime stimuli. This result strongly supports the null hypothesis that no effect of emotional priming was found when the prime stimuli was not experienced.

## 4 Discussion

In this paper, we have presented evidence suggesting that perception of emotional content involves multiple graded levels of awareness. Importantly, we have shown that degrees of awareness predict the effects of emotional priming, as well as facial expression recognition accuracy. The finding that the effects of priming is modulated by degree of conscious experience, and the result that there is no effect of emotional priming in the absence of conscious experience of the prime stimulus, together challenge what may be considered the “classical” interpretation of priming (Whalen, 1998; Morris et al., 1998; Esteves et al., 1994). According to the classical interpretation, priming can work unconsciously by modulating subsequent behaviour by way of implicit memory (Whalen, 1998; Morris et al., 1998; Esteves et al., 1994). The present finding, however, indicates that priming requires at least weak experience of the stimulus. This finding is in principle able to explain previous experimental results on priming, as priming has never before been investigated using a scale like PAS, which attempts to operationalise degrees of perceptual experience (Ramsøy and Overgaard, 2004). In that case, previous results from experiments on priming could at least in part represent cases of weak experiences.

Obviously, the conclusion may be limited to masked, emotional face priming, which theoretically might work differently than other kinds of priming. However, priming has previously been regarded as one, single phenomenon, investigated with different methods (see Windey et al., 2014 for a review), so in that case, this understanding should be revised. A recent study by Lähteenmäki et al. (2015) has shown that the ability to discriminate emotional stimuli of a range of different classes depends on the level of perceptual awareness. A finding which is supported by our 2AFC experiment. This could suggest that similar generalisability could be true for priming effects on emotional judgement. However, it remains to be seen whether such generalisability to different classes of stimuli can be applied to the priming effects on emotional judgement.

The conception that visual awareness has multiple levels has previously been demonstrated in behavioural and neuroimaging experiments with brief exposures of simple shapes and colours (Christensen et al., 2006, Ramsøy and Overgaard, 2004). Furthermore, “subliminal” recognition of words might be due to partial awareness of the priming words, and therefore also questions the argument that words can be subliminally recognised (Kouider and Dupoux, 2004, Fazekas and Overgaard, 2017). However, a recent study found that while perceptual awareness of simple visual features are most exhaustively described by a graded account of awareness, word stimuli are more adequately described by a dichotomous account of awareness (Windey et al., 2014). The finding that faces can be perceived with multiple levels of awareness, and that facial expression recognition is correlated with degree of awareness, provides further evidence for awareness as a graded phenomenon. It also supports the conception of perceptual awareness as graded for socially relevant stimuli. This finding enabled the investigations of the relationship between emotional priming and degree of awareness in the present study. Furthermore, it opens new doors for further investigations into the role of perceptual awareness of complex stimuli on behaviour.

Our interpretation above is based on the interpretation that participants are perceptually conscious when a participant reports *brief glimpse* of the prime stimulus, regardless of the ability to describe the actual content. Obviously, this rests upon one's preferred definition of consciousness. If one were to define perceptual experience so that a clear conception of the experienced object is required for that experience to be conscious, our results could in fact be taken to support the opposite view than what we are advocating here. That would, however, require a completely new conceptual category to speak about those first-person accessed states that have weak or no clearly defined object as its content. By large, scientists investigating consciousness define consciousness as subjective experience – anything that is accessed from a first person perspective. Accordingly, we believe that our interpretation of PAS is congruent with standard conceptions in the field.

Taken together, these results suggest that emotional priming of subsequent emotional judgements and facial expression recognition relates to *how clearly* a priming stimulus is perceived, and not just *if* it was perceived, as traditionally thought. By demonstrating a graded account of awareness in emotional priming, our study expands the growing body of evidence that questions the contributions of unconscious processing on behaviour, by providing evidence against, what has been considered, a robust demonstration of unconscious cognition for half a century.
