# Supplementary material for: Emotional priming depends on the degree of conscious experience
Source: Neuropsychologia. 2019 May;128:96–102. doi: 10.1016/j.neuropsychologia.2017.10.028 (PMC6562235; doi:10.1016/j.neuropsychologia.2017.10.028)
Supplement: Supplementary file 1 — Supplementary material [file mmc1.doc]

**Supplementary material for “Emotional priming depends on the degree of conscious experience”**

Michael Lohse and Morten Overgaard


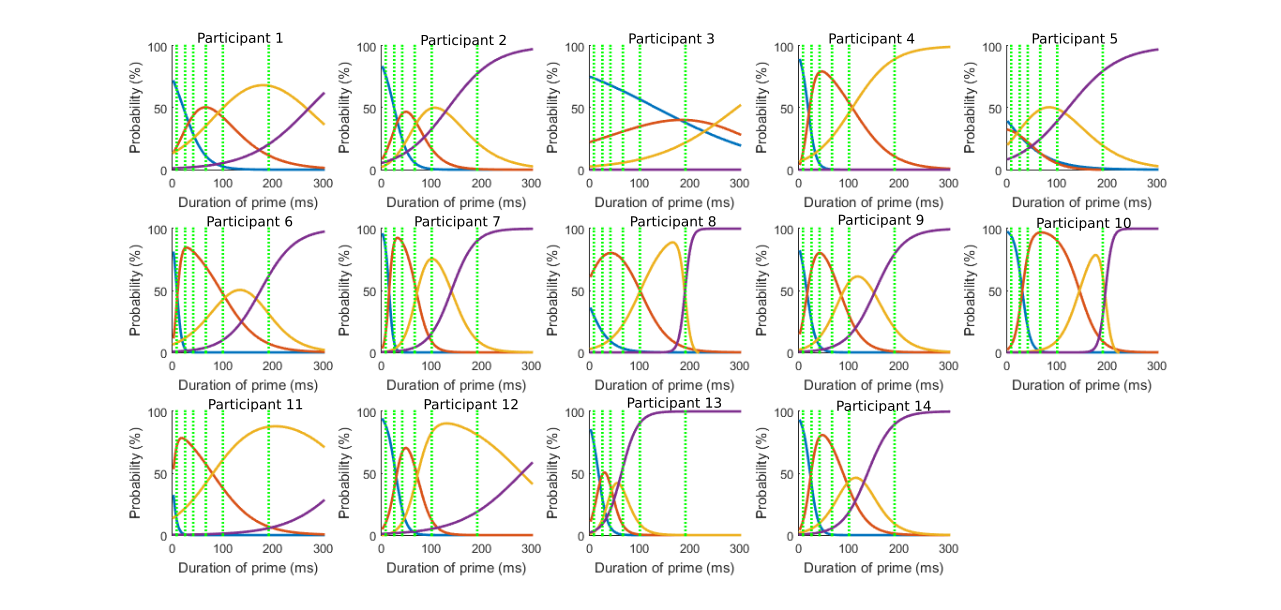


Figure *S1*: Within-subject multinomial regressions of probability of PAS values chosen, as a function of prime stimulus duration. *Blue: no experience, red: Brief Glimpse, Orange: Almost clear experience, Purple: Clear Experience.* Green lines indicate durations of prime stimuli presented.


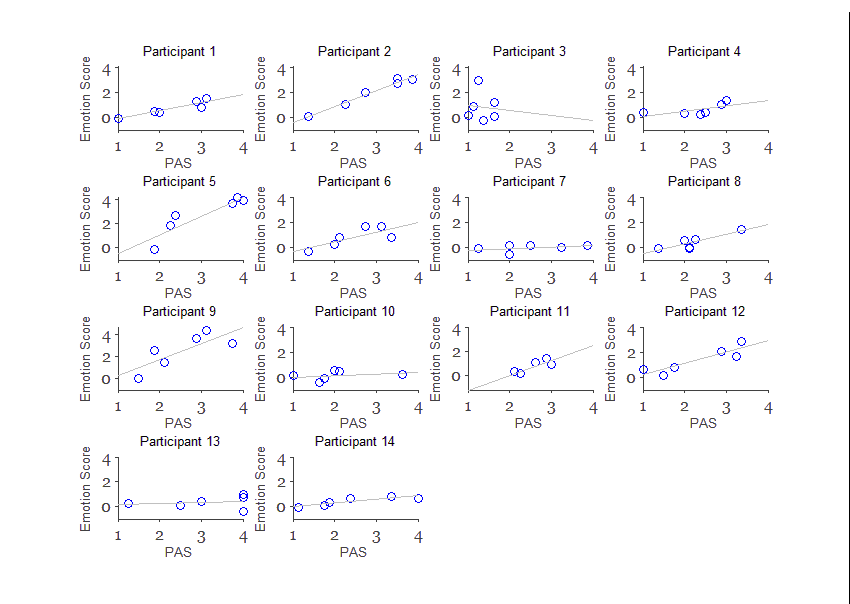


Figure *S2*. Within-subject linear regressions for positive emotional priming strength as a function of perceptual awareness. Each point is the mean PAS value for each presented prime stimulus duration. Emotion score is calculated by subtracting the SAM responses of the neutral priming stimuli from the SAM responses of the emotional priming stimuli at each stimulus duration. x*-*axis*:* *1 = No Experience, 2 = Brief Glimpse, 3 = Almost Clear Experience, 4 = Clear Experience.* The gray lines indicates least squares regression line.


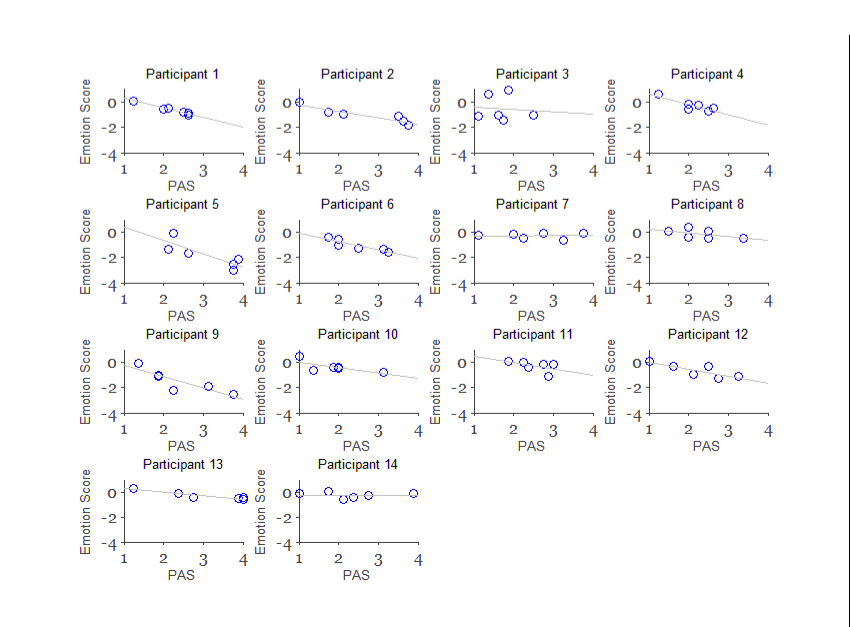


Figure *S3*. Within-subject linear regressions for negative emotional priming strength as a function of perceptual awareness. Each point is the mean PAS value for each presented prime stimulus duration. Emotion score is calculated by subtracting the SAM responses of the neutral priming stimuli from the SAM responses of the emotional priming stimuli at each stimulus duration. x-axis*:* *1 = No Experience, 2 = Brief Glimpse, 3 = Almost Clear Experience, 4 = Clear Experience..* The gray lines indicates least squares regression line.


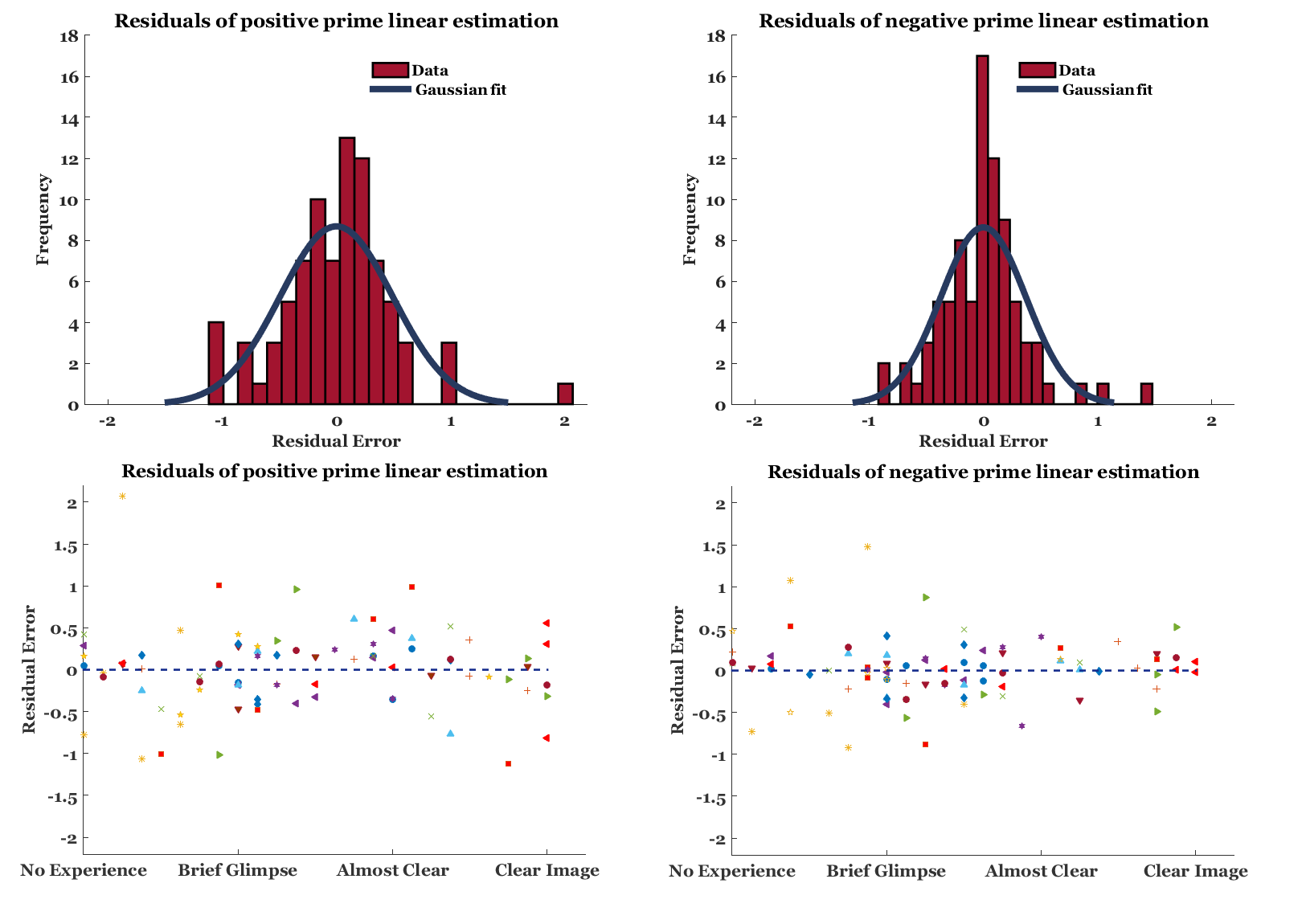


Figure *S4*: Distribution of residuals from concatenated within-subject linear regressions of emotional priming strength as a function of perceptual awareness. These results support that a linear approximation is a good model for describing the relationship between awareness and emotional priming. *Top)* Histogram of residuals for positive prime condition (left) and negative prime condition (right). *Bottom)* The distribution around the within subject regression lines for positive prime condition (left), and negative prime condition (right). Color/shape of points segregate residuals from individual participants (n = 14).
